# PITChing (professional organisations, innovative trial designs and collaborative approach) for evidence generation for proton therapy

**DOI:** 10.1186/s13014-020-01538-y

**Published:** 2020-06-01

**Authors:** Srinivas Chilukuri, Pankaj Kumar Panda, Rakesh Jalali

**Affiliations:** Apollo Proton Cancer Centre, Chennai, India

## Abstract

Developments in the field of proton beam therapy (PBT) have recently crossed the tipping point wherein the modality is now more versatile than ever before, with possibilities and likely indications expanding rapidly.

However the pace of evidence generation lags behind the developments in the field.

Generating quality evidence has its own set of challenges owing to complexities of conducting randomized controlled trials, which are the hallmark of level 1 evidence generation.

Here we discuss various challenges to clinical evidence generation in PBT and have suggested certain solutions including collaborative approaches and alternative study designs to mitigate these challenges.

Proton beam therapy (PBT) like other advances in oncology had its evolution spread across several decades. One might even say that progress in this field has been largely incremental. The obvious reasons for this are late adoption of this technology by hospitals, huge and expensive infrastructure requirement, cumbersome operations and relatively slower evidence generation. However, this field has gained momentum with the advent of spot scanning technology that led to several transformational changes that made PBT delivery more efficient and enabled the introduction of paraphernalia of the photon world, such as image guidance and motion management. This triggered better understanding of proton uncertainties, robust optimisation, better treatment planning algorithms, and several engineering advances including more efficient cyclotrons. Moving forward, proton therapy is likely to evolve into a more compact, robust and cost-effective technology.

## Challenges in evidence generation

Although there is a discerning lack of consensus regarding PBT’s benefits in several indications, it has been accepted as the preferred choice in paediatric cancers, complex spinal tumours, tumours requiring re-irradiation and certain CNS tumours with a reasonable consensus. For rest of the clinical indications it is widely believed that there is equipoise to merit randomised controlled trials (RCT). There are several ongoing RCTs comparing photons and protons in North America [1], and almost all of them are struggling to recruit patients. Several authors have articulated these challenges that are mainly relevant in the context of USA’s healthcare [2]. The central theme of all these concerns has been economics mainly in the form of lack of insurance coverage for several cancer sites. However, these set of challenges are likely to be different across regions globally. In most LMIC such as India, health care system is based on shared decision-making model and the financial standing of the patient dictates the decision regarding the use of PBT. Unfortunately, the funding opportunities in these countries are limited and the “RCT culture” (the drive to conduct or participate in trials) is restricted to a few institutions [3].

Much of the available evidence for PBT through RCTs have been obtained using the older passive scattering techniques which many believe will be rendered obsolete. Evaluation of evolving technologies is extremely challenging primarily because of resources and time constraints. By the time the results of the current RCTs are in public domain, the technology is likely to evolve and results may become irrelevant. So the RCTs from centres that have used treatment plans with relatively large spot sizes, no image guidance or large margins may not represent the true potential of modern PBT. Also, timing of a trial is extremely crucial as learnt from the RCT on non-small cell lung cancer [4]. The investigators noted a learning curve in treatment planning during the trial accrual, which could have altered the outcome of this study.

In the context of global and regional challenges in evidence generation for PBT, we would like to propose a definitive action plan to bring across major stakeholders such as governmental and non-governmental institutions in high-income countries (HIC) and LMIC, industry and professional organizations across the globe to address the challenges of evidence generation. The action plan proposed by us will be a multi-pronged approach involving relevant stakeholders, innovative study designs, potential funding mechanisms, and most importantly seamless coordination between all these factors to carry forward collaborative PBT trials to fruition by generation of high-quality evidence for PBT.

## Professional organisations role

### Advisory role to maintain practice quality standards and promote collaborative trial participation

Global organizations such as the NRG, NCI, NIH, RTOG, MRC, EORTC etc. have long recognized the need for multi-institutional global collaborative trials, to answer important clinical research questions. For PBT trials, the Particle Therapy Cooperative Group (PTCOG), Proton Collaborative group (PCG) or similar organisation can potentially become a nodal point providing funding opportunities, forging global partnerships to conduct clinical trials. Periodic audits and subsequent amendments of already ongoing studies can be undertaken by these central bodies based on evolving technologies and newly acquired information.

Essential credentialing for participation in multi-centric trials has always been a challenge in cross-country trials. Actively working alongside organisations such as the Radiological Physics Centre (also called the Imaging and Radiation Oncology Core [IROC] Trial Credentialing program) could possibly mitigate this challenge and result in effective utilisation of resources and faster credentialing processes.

### Funding for proton therapy trials

Institutional support for trials of advanced radiation oncology modalities, including proton therapy, is limited or extremely sparse. While in case of drug trials, pharmaceutical companies take a lead in providing funding and other resources, radiotherapy trials have rarely received such support. Forging a sustainable model for facilitating research with support from PBT vendors is definitely something to be worked upon.

### Data management and analytics

With data being already poised as valuable as currency, central organisations can act as data repository centres to facilitate generation of robust and effective patient models and data sets which can potentially have far reaching effects on clinical practices and subsequent outcomes.

## Innovative trial design

Clinical trial study designs and methodologies which enhance the value proposition of a trial with optimal sample size, reduced resource and capital expenditure with a concentric approach towards patient centric outcomes and quality of life are the need of the hour. Pragmatic clinical trial designs, adaptive designs, Bayesian methods, model-based approaches for patient selection, seeking biological surrogates for toxicities, risk-based monitoring, patient engagement during trial design etc. are steps in the right direction. Since most PBT trials need long follow-up periods, initiatives such as Intermediate Clinical Endpoints in Cancer of the Prostate (ICECaP) are much needed. Similarly, intermediate surrogates for late effects (ISLE)- should be sought to identify patients at higher risk to develop late effects [5]. Most or all of the next generation trials must include cost effectiveness (CE) analysis as the thresholds are constantly evolving.

## Collaborative approach

In LMIC, the overall cost of the proton treatment is likely to be a fraction of that in North America and Europe. Also, the cost of running a trial itself is much lower bringing down the overall funding requirement significantly lower compared to that in USA. Hence the output per dollar spent on clinical trials is far superior in LMIC. So economically speaking, it is prudent to fund a well-designed study in countries like India to quickly address a clinical question. Several institutions have previously funded major practice changing studies outside of North America [6] and the same standards can be applied to these studies. Investigators in HIC and emerging proton therapy centres in LMIC stand to benefit mutually by virtue of such collaborative proton therapy research. The HIC stand to benefit in terms of lower operational costs and timely completion of trials. While LMIC stand to gain in terms of financial assistance to conduct resource intensive clinical trials, faster evidence generation and eventually better utilisation of an expensive technology for deserving patients.

The barriers to evidence generation in PBT are sufficiently formidable however the way ahead is encouraging and can be accomplished so as to say. It requires concerted efforts from all the stakeholders across the globe. Each of the barriers here must be acknowledged and efforts made to overcome them. Unfortunately, it is fanciful to think this will happen any time soon.

## Data Availability

Not applicable.

## Abbreviations

PBT: Proton beam therapy
CNS: Central nervous system
USA: United States of America
RCT: Randomised controlled trials
HIC: High-income countries
LMIC: Low and middle income countries
NRG: RTOG has joined with national surgical adjuvant breast and bowel project (NSABP) and the gynaecologic oncology group (GOG) to form NRG oncology
NCI: National cancer institute
NIH: National institutes of health
RTOG: Radiation therapy oncology group
MRC: Medical research council, United Kingdom
EORTC: European organisation for research and treatment of cancer
PTCOG: Particle therapy cooperative group
PCG: Proton collaborative group
IROC: Imaging and radiation oncology core
ICECaP: Intermediate clinical endpoints in cancer of the prostate
ISLE: Intermediate surrogates for late effects
CE: Cost effectiveness
